# Genome-Wide Analysis of the PIN Auxin Efflux Carrier Gene Family in Coffee

**DOI:** 10.3390/plants9091061

**Published:** 2020-08-19

**Authors:** Xing Huang, Xuehui Bai, Tieying Guo, Zhouli Xie, Margit Laimer, Dengxiang Du, Thomas Gbokie, Zhirun Zhang, Chunping He, Ying Lu, Weihuai Wu, Kexian Yi

**Affiliations:** 1 Environment and Plant Protection Institute, Chinese Academy of Tropical Agricultural Sciences, Haikou 571101, China; hxalong@gmail.com (X.H.); hechunppp@163.com (C.H.); ytluy2010@163.com (Y.L.); yikexian@126.com (K.Y.); 2 Key Laboratory of Integrated Pest Management on Tropical Crops, Ministry of Agriculture and Rural Affairs, Haikou 571101, China; 3 Hainan Key Laboratory for Monitoring and Control of Tropical Agricultural Pests, Haikou 571101, China; 4 Dehong Tropical Agriculture Research Institute of Yunnan, Ruili 678600, China; 13529520059@163.com (X.B.); guotieying@126.com (T.G.); 5 School of Life Sciences, Peking University, Beijing 100871, China; xzhouli@pku.edu.cn; 6 Plant Biotechnology Unit, Department of Biotechnology, BOKU-VIBT, University of Natural Resources and Life Sciences, Vienna 1190, Austria; 7 National Key Laboratory of Crop Genetic Improvement, Huazhong Agricultural University, Wuhan 430070, China; ddx@mail.hzau.edu.cn; 8 College of Plant Protection, Nanjing Agricultural University, Nanjing 210095, China; gbokiejr@gmail.com; 9 Coffee Engineering Research Center of China, Mangshi 678400, China; zhizhi9752@163.com

**Keywords:** PIN, auxin efflux carrier, phylogeny, expression, *Coffea arabica*, *Coffea canephora*, *Coffea eugenioides*

## Abstract

Coffee is one of the most popular beverages around the world, which is mainly produced from the allopolyploid *Coffea arabica*. The genomes of *C. arabica* and its two ancestors *C. canephora* and *C. eugenioides* have been released due to the development of next generation sequencing. However, few studies on *C. arabica* are related to the PIN-FORMED (PIN) auxin efflux transporter despite its importance in auxin-mediated plant growth and development. In the present study, we conducted a genome-wide analysis of the PIN gene family in the three coffee species. Totals of 17, 9 and 10 of the PIN members were characterized in *C. Arabica*, *C. canephora* and *C. eugenioides*, respectively. Phylogenetic analysis revealed gene loss of PIN1 and PIN2 homologs in *C. arabica*, as well as gene duplication of PIN5 homologs during the fractionation process after tetraploidy. Furthermore, we conducted expression analysis of *PIN* genes in *C. arabica* by in silico and qRT-PCR. The results revealed the existence of gene expression dominance in allopolyploid coffee and illustrated several PIN candidates in regulating auxin transport and homeostasis under leaf rust fungus inoculation and the tissue-specific expression pattern of *C. arabica*. Together, this study provides the basis and guideline for future functional characterization of the PIN gene family.

## 1. Introduction

Auxin is one of the most important phytohormones and plays a critical role in regulating plant growth and development [1]. As the main form of auxin, indole-3-acetic acid (IAA) can be passively transported in phloem in a fast non-directional manner [2]. There is also a slow and directional polar auxin transport (PAT) between cells, which is mediated by auxin influx and efflux carriers [3]. Among these, members of the PIN_FORMED (PIN) family have been well-characterized for their function as auxin efflux carriers [4]. PAT triggers auxin gradient distribution to form maxima, which is closely related with tissue development [2]. In *Arabidopsis*, eight PINs have been reported with two distinct subcellular localizations. For instance, five PINs (*AtPIN1–4* and *7*) are located at the plasma-membrane, linking their functions with the cell-to-cell PAT [5]. *AtPIN5* and *8* reside within the endoplasmic reticulum (ER), manipulating the cellular auxin homeostasis [6,7]. Interestingly, *AtPIN6* is targeted to both plasma membrane and ER, implying that it might mediate cell-to-cell PAT and cellular auxin homeostasis [8]. To date, the eight *Arabidopsis* PINs have been functionally characterized in regulating plant development. *AtPIN1–3* and *7* are directly related to the gravitropic response regarding their PAT function [9,10,11,12]. *AtPIN1*, *3*, *4* and *7* are involved in phototropic responses [13,14]. Usually, *AtPINs* are expressed in auxin-mediated fast developmental tissues. *AtPIN1* has multiple functions in regulating floral bud formation, leaf shape and vein patterning [15,16]. *AtPIN2* is mainly expressed in cortical and epidermal cells during root tip elongation [17,18]. *AtPIN3* regulates apical hook formation/maintenance and lateral root formation [19,20]. *AtPIN4* is necessary for apical hook development and root patterning [14,21]. *AtPIN5* is involved in the initiation of the lateral root, the growth of root and hypocotyl, and the expansion of cotyledon, based on analysis of PIN5 overexpressing lines [7]. *AtPIN6* obtains more functions than other PIN members, such as regulating shoot apical dominance, the development of adventitious root, lateral root primordia, root hair, inflorescence stem, short stamen and nectar [8,22,23]. *AtPIN7* controls the development of early embryogenesis and the apical hook [2,14]. *AtPIN8* is specifically expressed in pollen, regulating sporophyte and male gametophyte development [5,6,24].

Nowadays, due to the development of next-generation sequencing technology, more and more plant genomes have been assembled and released, which makes genome-wide analysis of the PIN family successful [25]. In total, there are 8, 17, 12, 11, 14, 10, 15, 10, 11, 23 and 20 PIN members characterized in the genomes of *Arabidopsis thaliana*, cotton (*Gossypium hirsutum*), maize (*Zea mays*), *Medicago truncatula*, pear (*Pyrus bretschneideri*), pepper (*Capsicum annuum*), poplar (*Populus trichocarpa*), potato (*Solanum tuberosum*), *Sorghum bicolor*, soybean (*Glycine max*) and tobacco (*Nicotiana tabacum*), respectively [4,26,27,28,29,30,31,32,33,34,35]. However, few PIN-related studies are reported in coffee species, despite the fact that it is one of the most popular beverages around the world. In the present study, we carried out a genome-wide analysis of the PIN family in the allotetraploid *Coffea arabica* and its two diploid ancestors, *C. canephora* and *C. eugenioides*. Phylogenetic, structural and gene expression analyses were also conducted to reveal the evolution and expression patterns of the coffee PIN family. Together, our results can provide a guideline for future coffee PIN gene-related studies.

## 2. Results

### 2.1. Genome-Wide Identification of PIN in Coffee Species

Eight *Arabidopsis* PIN protein sequences were selected to search against the three coffee genomes (Table S1) [4]. The results showed that 17 PINs (*CaPINs*) were found in the *Coffee arabica* genome and 10 in *C. canephora* (*CcPINs*) and *C. eugenioides* (*CePINs*) (Table 1), in which *CaPINs* were located on 10 chromosomes and 1 unanchored scaffold with coding sequence (CDS) lengths of about 1074–1995 bp, proteins lengths about 357–664 aa, molecular weight of about 38,862.37–71,668.43 and isoelectric point (pI) of about 6.95–9.11. In *Coffea canephora*, the *CcPINs* were mapped on seven chromosomes with CDS length, protein length, molecular weight and theoretical isoelectric points (pI) ranging from 444–1998 bp, 147–665 aa, 16,560.9–71,838.64 and 7–9.3, respectively. *CePINs* were located on five chromosomes and one unanchored scaffold, and their four characters varied between 1074–1986 bp, 357–661 aa, 38,879.22–71,315.05 and 6.95–9.05, respectively. Moreover, all *Arabidopsis* and coffee PINs were predicted to be located at the plasma membrane (Table 1, Table S1). CELLO also predicts endomembrane-localized *Arabidopsis thaliana* PINs to localize to the plasma membrane. Therefore, the exact cellular localization (plasma membrane or endomembrane) would require experimental validation. These proteins also contained 5–10 transmembrane helices at the N- and C-termini (Figure S1).

### 2.2. Phylogenetic Analysis of Coffee PIN

**Figure 1:**
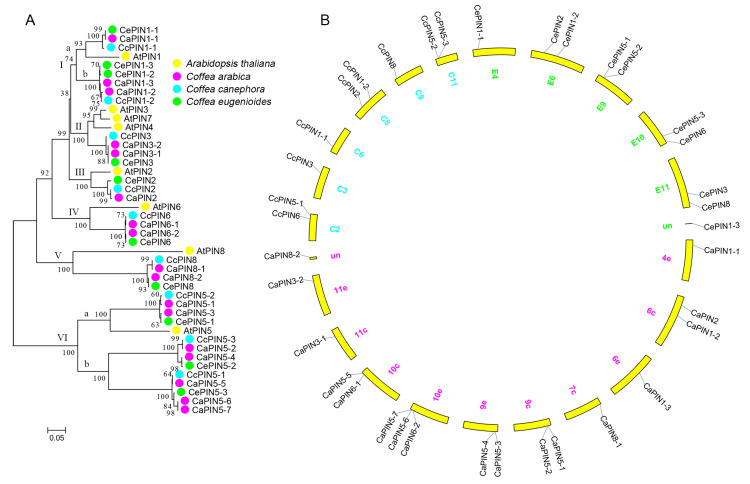
Phylogenetic (**A**) and chromosomal distribution (**B**) of the coffee PIN family. The protein sequences of *Arabidopsis thaliana*, *C. arabica*, *C. canephora* and *C. eugenioides* are shown in yellow, pink, blue and green, respectively (A). The chromosome numbers are listed beside chromosomes (yellow) for *C. arabica* (pink), *C. canephora* (blue) and *C. eugenioides* (green). The *un* represents the unanchored scaffold.

The protein sequences of *Arabidopsis* and coffee PIN were selected for phylogenetic analysis. A total 44 of AtPINs/CaPINs/CcPINs/CePINs were separated into six groups (Figure 1A). Three PIN of *C. arabica* and *C. eugenioides* were in group I together with two from *C. canephora* and one from *Arabidopsis*. Group II contained three PIN of *Arabidopsis*, two of *C. arabica* and one of each ancestor. In group III–V, there were two members of *C. arabica*, one of each ancestor and *Arabidopsis*. Besides, seven, three, three and one PIN(s) were from *C. arabica*, *C. canephora*, *C. eugenioides* and *Arabidopsis*, respectively. A chromosomal distribution map was also constructed according to the chromosomal locations of coffee *PIN genes* (Table 1, Figure 1B). Additionally, coffee PINs were further aligned to examine their conserved domains, and it was observed that they contained two conserved domains at the N- and C-termini (Figure S2). We further calculated the nonsynonymous (Ka) and synonymous nucleotide substitutions (Ks) and their ratio (Ka/Ks) among the coffee *PIN* gene pairs (Table S2). Apparently, none of the gene pairs was subjected to positive selection (Ka/Ks > 1), suggesting the conserved evolution of coffee *PIN* genes.

### 2.3. Cis-Element Prediction and Gene Structure Analysis of Coffee PIN

**Figure 2:**
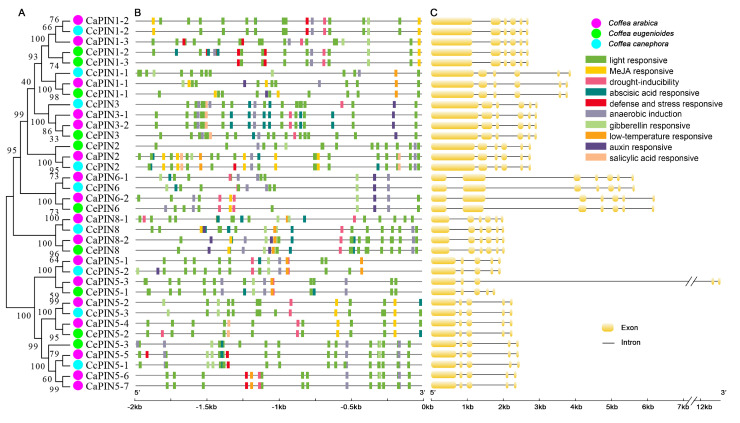
*Cis*-element (**A**) and intron (**B**) analyses of the coffee PIN family. The *cis*-element analysis was conducted with 2kb upstream from the initiation codon using PlantCARE software. The intron analysis was carried out with genomic and coding sequences of the coffee PIN family.

The upstream sequences (2000 bp) of coffee *PINs* were analyzed for *cis*-elements (Table S3). Ten regular *cis*-regulatory elements, including elements related to anaerobic induction, light response, gibberellin response, methyl jasmonate (MeJA) response, abscisic acid response, defense and stress response, drought-inducibility, low-temperature response, salicylic acid response and auxin response, were annotated (Figure 2A). Moreover, intron analyses indicated that most of *PINs* contain five or six introns (Figure 2B). Surprisingly, *CaPIN5-3* contained an intron of more than 10 kb in length.

### 2.4. In Silico Expression of Coffee PINs under Biotic Stress

**Figure 3:**
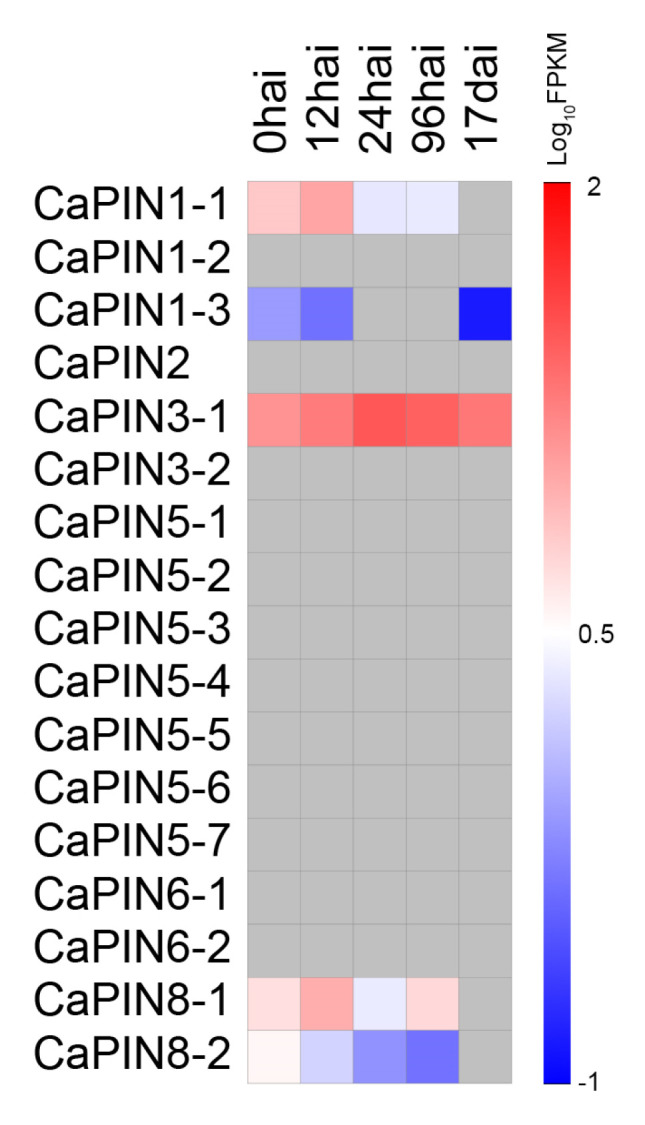
In silico expression of *PINs* in the leaf of *C. arabica* after inoculation with the coffee leaf rust fungus *Hemileia vastatrix*. Hai and dai represent hours and days after inoculation, respectively. Grey squares represent no data.

To evaluate the expression pattern of *PINs* under biotic stress, in silico analyses was carried out with inoculation of the coffee leaf rust fungus *Hemileia vastatrix*. The results showed that 5 of the 17 genes were regulated under stress condition (Figure 3). Among these, three were up-regulated and afterwards down-regulated (*CaPIN1-1*, *CaPIN3-1* and *CaPIN8-1*), while the other two were continuously down-regulated during the entire process (*CaPIN1-3* and *CaPIN8-2*).

### 2.5. Expression Profiles of Coffee PINs in Different Coffee Tissues

**Figure 4:**
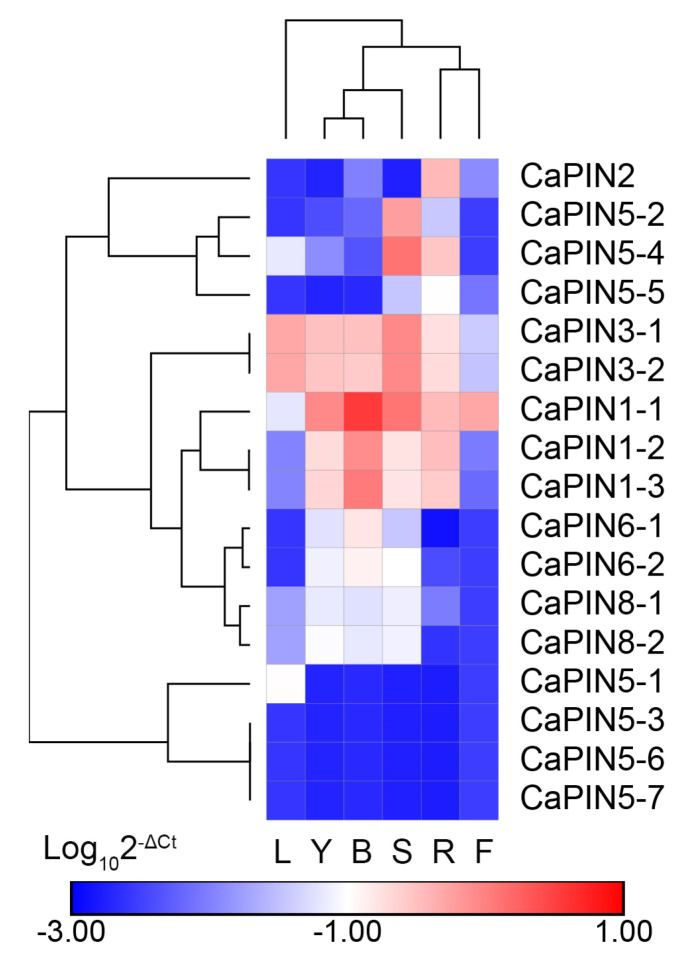
Expression pattern of PIN genes in different tissues of *C. arabica* determined by qRT-PCR: L, Y, B, S, R and F represent mature leaf, young leaf, bud, stem, root and fruit, respectively.

We further examined the expression of *PINs* in different tissues of *C. arabica* by qRT-PCR (Figure 4, Table S4). The results indicate that *CaPIN1-1* was highly expressed in young leaves, roots, stems and buds. *CaPIN1-2* and *CaPIN1-3* shared a similar expression pattern and were highly expressed in buds. *CaPIN2* was mainly expressed in roots. *CaPIN3-1* and *CaPIN3-2* showed similar expression patterns with relatively high expression levels in all five tissues except the fruits. However, *CaPIN3-2* and *CaPIN5-3*/*6*/*7* displayed an extremely low expression level in all tested tissues. *CaPIN5-1* was mainly expressed in leaves, but at a low level. *CaPIN5-2*/*4* showed a high expression in stems. *CaPIN6-1*/*2* and *CaPIN8-1*/*2* were mainly expressed in young leaves, buds and stems with similar patterns.

## 3. Discussion

### 3.1. Evolution of the Coffee PINs

In the present study, 17 *PINs* were characterized in *C. arabica*, while 9 and 10 members were identified in its two ancestors *C. canephora* and *C. eugenioides*, respectively (Table 1). *C. arabica* is a self-compatible perennial allotetraploid species derived from a spontaneous hybridization between the two closely related diploid ancestors. Theoretically, there should be 20 PIN members in the *C. arabica* genome. According to the phylogenetic results, the numbers in group II, IV, V and VIa proved the theory (Figure 1A). Interestingly, the three coffee species share the same number of PINs in group Ia and III. Moreover, *C. arabica* contains five PIN members in group VIb, twice more than the numbers of each ancestor, which might be due to the complex genome rearrangements during the generation of *C. arabica*, even if its divergence time (0.665 million years) is considerably shorter than that between the two ancestors (4.2 million years) [36]. This phenomena of gene loss in group I and III is closely related with a fractionation bias, which has been reported in *Arabidopsis* and maize [37,38]. The gene loss is most likely due to bulk DNA removal induced by intra-chromosomal recombination [38]. After reaching tetraploidy, the increased chromosome number has also been described to promote the frequency of chromosome recombination [39]. Several chromosomes of the C subgenome in *C. arabica* are significantly longer than those in *C. canephora* (Table 1, Figure 1B). Furthermore, the promoter and coding regions of the coffee PIN genes were affected by this process (Figure 2). Nucleotide sequence changes in promoter regions directly contribute to gene expression [40], and these changes occurring in coding regions will directly alter gene function [41]. Moreover, the neighbor gene pair *CaPIN5-6*/*7* with the same CDS is a duplication event during the process of fractionation (Figure 1, Table S2). This expansion might be caused by gene cassette translocation, which is different from early duplication events accompanied with the divergence of a genus or a family [42]. In coffee genomes, the PIN1 and PIN5 groups are expanded when compared with *Arabidopsis* (Figure 1). The expansion of the PIN1 group is commonly observed in other plants, such as cotton, grasses, pear, pepper, poplar, potato, soybean and tobacco [27,28,29,31,32,33,34,35]. In contrast, the PIN5 group is expanded in grasses and tobacco [31,33]. *AtPIN1* and *AtPIN5* are defining members of two functionally divergent groups PIN1 directional polar auxin transport and PIN5 regulation of auxin transport between cytosol and ER [5,6,7]. The expansion of these two genes might contribute to auxin-mediated plant development and environmental adaptation [43].

### 3.2. Candidate Regulators in the Coffee PIN family

Our in silico analysis revealed that there are five coffee PINs that are regulated after fungal inoculation (Figure 3). For instance, *CaPIN1-1* and *CaPIN8-1* reach a peak expression at 12 hai and *CaPIN3-1* at 24 hai, which might be caused by plant cell wall-mediated immunity [44]. Moreover, the diverse expression of *CaPIN1-1*/*2*/*3* and *CaPIN3-1* indicate that they might function as main auxin polar transporters in coffee tissues (Figure 4). The peak expression of *CaPIN2* in roots and *CaPIN5-2*/*4* in stems suggests that they are the good candidates in regulating root and stem development, respectively. The results also indicate that *CaPIN6-1*/*2* and *CaPIN8-1*/*2* might control auxin homeostasis in young leaves, stems and buds. In addition, *CaPIN1-2*/*3*, *CaPIN6-1*/*2* and *CaPIN8-1*/*2* display slightly different expression patterns within the three gene pairs, indicating the existence of gene expression dominance in allopolyploid coffee that might be controlled by *cis*-elements [45]. The promoter regions showed significant differences within the three gene pairs (Figure 2), implying that *PIN* expression might be regulated at the transcription level. In addition, three kinds of *cis*-elements were identified in the promoter regions of all coffee PIN genes, including anaerobic induction, light response and drought-inducibility, indicating that all coffee *PINs* might be involved in these stresses responses. The other seven kinds of *cis*-elements were separately identified in different parts of coffee *PINs*, which might contribute to their different response to phytohormone and stress signals. Besides, the existence of a duplicated gene pair (*CaPIN5-6*/*7*) indicates the redundancy and functional loss across the PINs family. However, more evidence is still needed to reveal the mechanisms between duplication and fractionation after reaching tetraploidy of coffee.

## 4. Materials and Methods

### 4.1. Sequence Retrieval and Phylogenetic Analysis

The protein sequences of eight *Arabidopsis* PINs were selected to search against the genomes of *C. arabica*, *C. canephora* and *C. eugenioides* in the NCBI Genome Database by Tblastn method [4,46,47]. Obtained coffee PIN genes were named according to their *Arabidopsis* homologs and chromosome positions. Their accessions and chromosome locations are listed in Table 1. The protein characteristics, including lengths, molecular weights, and pI, were online predicted by ProtParam tool [48]. Subcellular localization was examined by CELLO software [49]. The TMHMM Server was used to analyze protein transmembrane topology [50]. Phylogenetic analysis was carried out with MEGA7 software [51]. A neighbor-joining (NJ) tree was constructed with bootstrap values tested for 1000 trails. DNAMAN 7 software was used to find the conserved domains of coffee PIN proteins and calculate sequence identities of the coffee PIN gene pairs [52]. Ka, Ks and Ka/Ks values were calculated by using DnaSP5.0 [53]. Ka/Ks > 1 represents positive selection and Ka/Ks ≤ 1 represents purified selection [54]. A chromosomal distribution map was constructed by TBtools [55].

### 4.2. Cis-Element and Gene Structure Analysis

The promoter (2 Kb upstream of the initiation codon) and genomic sequences of coffee PIN genes were retrieved from the three coffee genomes, respectively. The promoter sequences were upload to the online software PlantCARE for identifying putative *cis*-elements associated with growth, development and stress responses under default settings [56]. The image data were displayed in TBtools [55].

### 4.3. Plant Materials and RNA Extraction

The plants of *C. arabica* cultivar Bourbon were planted in the germplasm garden of Dehong Tropical Agriculture Research Institute of Yunnan (24.02° N, 97.85° E). Leaf, bud and flower samples were separately collected from 3-year-old plants at the flowering stage in March. Fruit samples were collected 2 months after flowering. Young leaf, stem and root samples were collected from seedlings with a height of 10 cm. Each sample was collected for three times from different plants as biological replicates and immediately placed into liquid nitrogen. RNA samples were extracted by Tiangen RNA prep Pure Plant Kit (Tiangen Biomart, Beijing, China) according to the manufacturer’s protocol and then stored at −80 °C.

### 4.4. Expression Analysis

For in silico expression analysis, the raw read data by RNA-seq were downloaded from the Sequence Read Archive (SRA) database under the accession of PRJNA353185 [57]. After trimming, the clean reads were mapped to *CaPINs* and the read count for each gene was obtained by RSEM software [58]. All read counts were normalized into fragments per kilobase per million mapped reads (FPKM).

For qRT-PCR expression analysis, RNA samples were reverse transcribed by using the GoScript Reverse Transcription System (Promega, Madison, WI, USA). Each qRT-PCR reaction contained a final volume of 20 μL including 1 μL cDNA template, 0.5 μL gene-specific primers (10 μM), 10 μL TransStart Tip Green qPCR Supermix (Transgen Biotech, Beijing, China), 0.4 μL Passive ReferenceDye (50×) (Transgen Biotech, Beijing, China) and 7.6 μL ddH2O. qRT-PCR reaction was conducted with a QuantStudio 6 Flex Real-Time PCR System (Thermo Fisher Scientific, Waltham, MA, USA). The thermal cycles were 94 °C for 30 s, 40 cycles of 94 °C for 5 s and 60 °C for 30 s, and were followed by a dissociation stage. Each sample was repeated three times as technical repeats. Specific primers for *CaPINs* were designed with Primer 3 (Table 2) [59]. Coffee *protein phosphatase* and *tubulin* genes were selected as endogenous control according to a previous study [60]. The ΔΔCt method was used for calculating relative expression levels [61].

## Figures and Tables

**Table 1:** Gene ID, accession, sequence length, molecular weight, theoretical isoelectric points (pI) and CELLO localization of coffee *PINs*.

ID	NCBI Accession	Chromosome Location	Coding Sequence (bp)	Predicted Protein (aa)	Molecular Weight	pI	CELLO Localization
CaPIN1-1	LOC113742499	Chr4e:5319931-5324277(+)	1809	602	66,068.52	8.78	PlasmaMembrane (4.325)
CaPIN1-2	LOC113692256	Chr6c:19755675-19759117(−)	1800	599	64,648.69	9.11	PlasmaMembrane (4.541)
CaPIN1-3	LOC113695379	Chr6e:18475803-18479240(−)	1800	599	64,660.77	9.06	PlasmaMembrane (4.613)
CaPIN2	LOC113691530	Chr6c:8677641-8680633(+)	1857	618	67,154.96	9.04	PlasmaMembrane (4.413)
CaPIN3-1	LOC113716800	Chr11c:28762761-28766913(+)	1995	664	71,668.43	7.71	PlasmaMembrane (3.822)
CaPIN3-2	LOC113719107	Chr11e:36778779-36782931(+)	1995	664	71,668.43	7.71	PlasmaMembrane (3.822)
CaPIN5-1	LOC113708749	Chr9c:3275956-3277726(+)	1074	357	39,130.15	7.66	PlasmaMembrane (4.154)
CaPIN5-2	LOC113708750	Chr9c:3294659-3297259(+)	1077	358	39,279.26	7	PlasmaMembrane (4.359)
CaPIN5-3	LOC113709610	Chr9e:2787073-2799648(+)	1074	357	39,084.12	7.66	PlasmaMembrane (4.072)
CaPIN5-4	LOC113709696	Chr9e:2809701-2812402(+)	1077	358	39,286.23	6.95	PlasmaMembrane (4.384)
CaPIN5-5	LOC113714408	Chr10c:43832409-43834939(+)	1089	362	40,211.78	7.99	PlasmaMembrane (4.578)
CaPIN5-6	LOC113712046	Chr10e:38622577-38625089(+)	1089	362	40,315.95	7.99	PlasmaMembrane (4.577)
CaPIN5-7	LOC113712037	Chr10e:38697672-38700185(+)	1089	362	40,315.95	7.99	PlasmaMembrane (4.577)
CaPIN6-1	LOC113713240	Chr10c:42560611-42566696(+)	1635	544	59,358.58	8.67	PlasmaMembrane (4.589)
CaPIN6-2	LOC113710898	Chr10e:37414288-37421605(+)	1644	547	59,627.88	8.52	PlasmaMembrane (4.621)
CaPIN8-1	LOC113698547	Chr7c:901163-904039(−)	1080	359	38,908.26	9.02	PlasmaMembrane (4.057)
CaPIN8-2	LOC113722850	NW_020850478.1:2088684-2091128(−)	1080	359	38,862.37	8.89	PlasmaMembrane (3.982)
CcPIN1-1	Cc04_g06290	Chr6:4778381-4782838(+)	1809	602	66,017.47	8.78	PlasmaMembrane (4.238)
CcPIN1-2	Cc06_g19880	Chr8:22061777-22064841(−)	1800	599	64,662.72	9.11	PlasmaMembrane (4.560)
CcPIN2	Cc06_g12940	Chr8:10631952-10634716(+)	1857	618	67,168.99	9.04	PlasmaMembrane (4.450)
CcPIN3	Cc11_g08680	Chr3:26054971-26059237(+)	1998	665	71,838.64	7.71	PlasmaMembrane (3.852)
CcPIN5-1	Cc10_g14830	Chr2:25683429-25685861(+)	1089	362	40,211.78	7.99	PlasmaMembrane (4.578)
CcPIN5-2	Cc09_g03470	Chr11:2994318-2996247(+)	1074	357	39,132.12	7.66	PlasmaMembrane (4.147)
CcPIN5-3	Cc09_g03480	Chr11:3012816-3015442(+)	1077	358	39,279.26	7	PlasmaMembrane (4.359)
CcPIN6	Cc10_g12950	Chr2:22666072-22671714(−)	1635	544	59,372.6	8.67	PlasmaMembrane (4.596)
CcPIN8	Cc07_g03020	Chr9:2053274-2055299(−)	1080	359	38,908.26	9.02	PlasmaMembrane (4.057)
CePIN1-1	LOC113768376	Chr4:4999933-5004212(+)	1809	602	66,068.52	8.78	PlasmaMembrane (4.325)
CePIN1-2	LOC113775722	Chr6:26100829-26104135(−)	1800	599	64,602.73	9.05	PlasmaMembrane (4.549)
CePIN1-3	LOC113759196	NW_020864866.1:1155-4437(+)	1800	599	64,616.75	9.05	PlasmaMembrane (4.566)
CePIN2	LOC113776283	Chr6:12410330-12413098(+)	1845	614	66,772.54	9.03	PlasmaMembrane (4.414)
CePIN3	LOC113751527	Chr11:38612643-38616661(+)	1986	661	71,315.05	7.71	PlasmaMembrane (3.858)
CePIN5-1	LOC113783693	Chr9:5448811-5450798(+)	1074	357	39,098.15	7.66	PlasmaMembrane (4.085)
CePIN5-2	LOC113783723	Chr9:5460896-5463464(+)	1077	358	39,300.26	6.95	PlasmaMembrane (4.393)
CePIN5-3	LOC113750662	Chr10:31456928-31459384(−)	1089	362	40,293.93	7.99	PlasmaMembrane (4.574)
CePIN6	LOC113750213	Chr10:36854392-36860603(−)	1620	539	59,223.7	8.64	PlasmaMembrane (4.611)
CePIN8	LOC113754254	Chr11:48522325-48524966(+)	1080	359	38,879.22	9.02	PlasmaMembrane (3.985)

**Table 2:** Primers used for qRT-PCR analysis.

ID	Forward Primer	Reverse Primer	Product Length
*CaPIN1-1*	TTTTGATGGGGCTATGAAGC	TCTCTGGACTCCATCCATCC	155
*CaPIN1-2*	GGGTATCCGGATCAATCCTT	AATTGTTTGCCCTCATGGAC	173
*CaPIN1-3*	AGGCAATCCCAGGGACTTAT	TTTTGGCCTTTTGGGTGTAG	211
*CaPIN2*	GTTCAGGCATCAACCGTTTT	TTCCAAGCTTCCATTTTTGG	182
*CaPIN3-1*	GGTTGAGTCCGACGTTGTTT	ACCCTCTTGGCGTAGGATTT	221
*CaPIN3-2*	TGGCTCTTCAGCCAAAGATT	TTTGGCAAACACAAATGGAA	191
*CaPIN5-1*	TATTGCGATGTATTAGTACACC	GTACTGTTGAAGAGGTGTTCCG	141
*CaPIN5-2*	GCCCTTTAATCCACAACACG	ATCGAATGCTCTTCCCGATA	238
*CaPIN5-3*	ATTCGAAATAAAAGTGGTGT	TTACTTTGAGTGGATAGCAA	199
*CaPIN5-4*	TCACTACAAATAACAATTGCT	GTAGCTTATATTGTAAAGTGT	153
*CaPIN5-5*	AATTATTTTACCCTTTCCTCC	AAATGAGGCTATGGATTGTGG	175
*CaPIN5-6*	TAGGAGGGGGCCAATTTTAT	TAAACCACGGCAGGCTAAGT	180
*CaPIN5-7*	TGCATAGCTGGCTTGATTTG	CTTAAAGCCATCCCGAACAA	197
*CaPIN6-1*	TGTAGTATACGTAACACGCTA	ACTTTATTATTCAACCCAACC	146
*CaPIN6-2*	TGTCGTTCTCTTTTGTGGTTG	GGAAGGAAGCCAACAAAGGAC	205
*CaPIN8-1*	AAATTGTCGCCGAATTTCAC	TGCACCAAATTTATCGTTCG	196
*CaPIN8-2*	TGAAGACAGGAGGTGTCGTG	GGATTGCCAAATCTGCATCT	225
*Protein phosphatase*	ATGTGGACCGAGGAAAACAG	AGGGCAGCTACAGGAAGACA	214
*Tubulin*	AAGTACTCCGGCGACTCAGA	GGCGGAAGATCTGACCATAA	157

## Supplementary Materials

The following are available online at https://www.mdpi.com/2223-7747/9/9/1061/s1, Figure S1: Transmembrane topology analysis of coffee PIN proteins. Figure S2: Conserved domains of coffee PIN proteins. Table S1: Gene ID, accession, sequence length, molecular weight, pI and CELLO localization of Arabidopsis *PINs*. Table S2: Sequence identity, nonsynonymous (Ka), synonymous nucleotide substitutions (Ks) and the ratio (Ka/Ks) of the coffee PIN gene pairs. Table S3: *Cis*-element prediction in upstream sequences of coffee PIN genes. Table S4: Ct values of *CaPINs* in different samples.
