# Supplementary figures and images for: Genome-Wide Analysis of the PIN Auxin Efflux Carrier Gene Family in Coffee
Source: Plants (Basel). 2020 Aug 19;9(9):1061. doi: 10.3390/plants9091061 (PMC7570243; doi:10.3390/plants9091061)

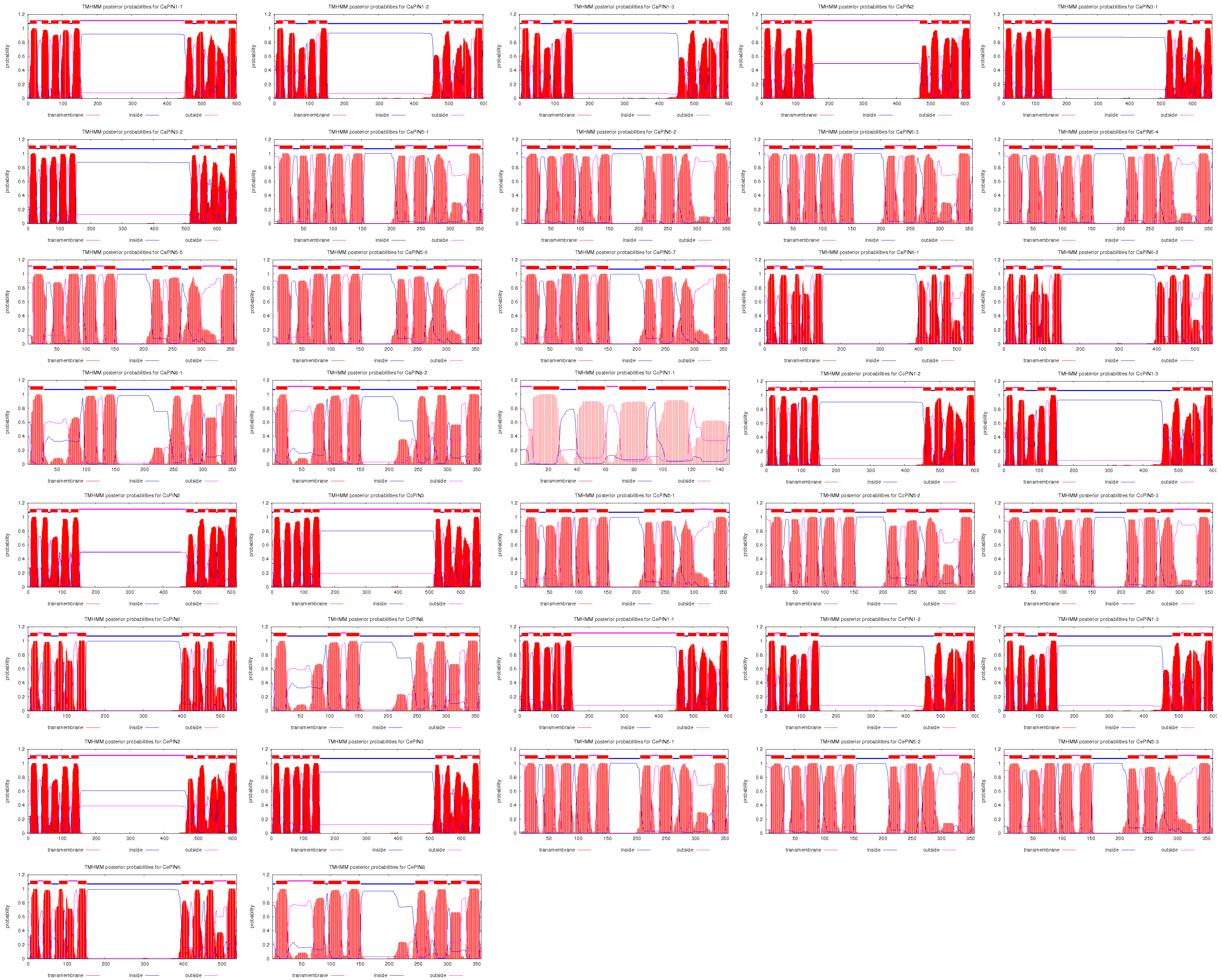

Supplement: Supplementary file 1 [file plants-09-01061-s001.zip › supplementary/Figure S1.pdf]

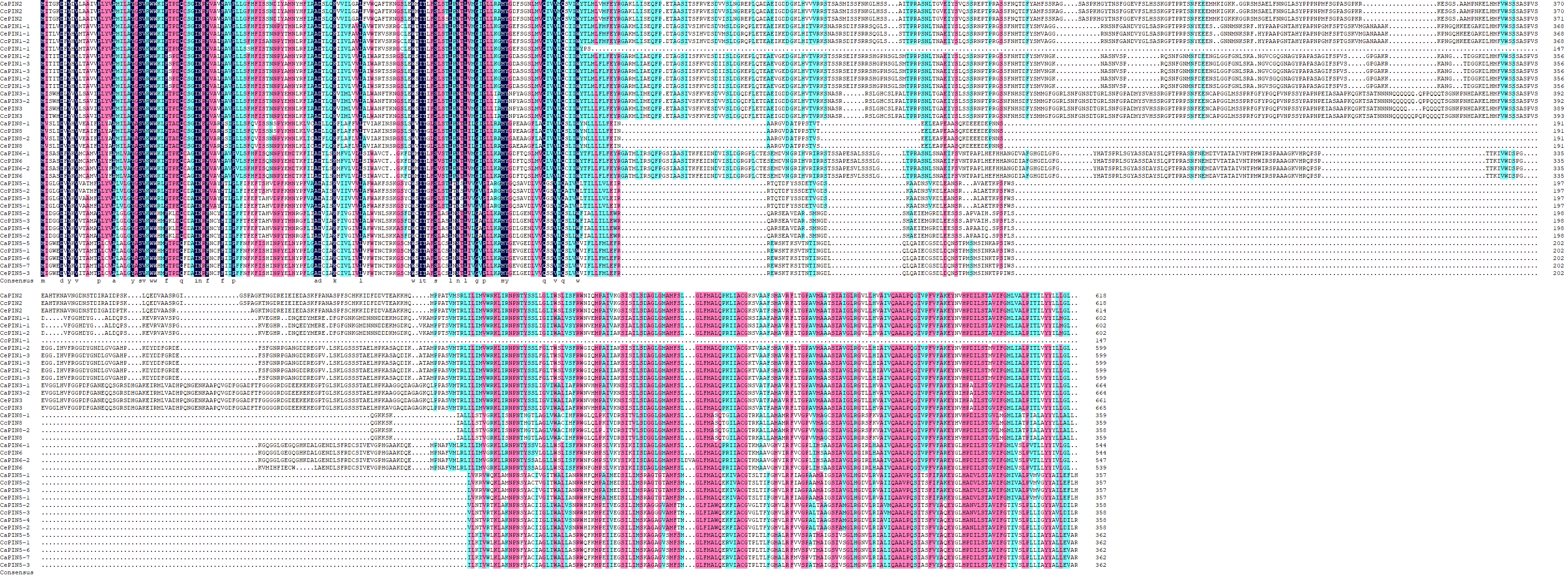

Supplement: Supplementary file 1 [file plants-09-01061-s001.zip › supplementary/Figure S2.bmp]
